# Mutational optimization of the coelenterazine-dependent luciferase from *Renilla*

**DOI:** 10.1186/1746-4811-4-23

**Published:** 2008-09-30

**Authors:** Jongchan Woo, Albrecht G von Arnim

**Affiliations:** 1 Department of Biochemistry, Cellular and Molecular Biology, The University of Tennessee, Knoxville, TN 37996-0840, USA; 2 The Rockefeller University, Molecular, Cell and Developmental Biology, 1230 York Avenue New York, NY 10065, USA

## Abstract

*Renilla *luciferase (RLUC) is a popular reporter enzyme for gene expression and biosensor applications, but it is an unstable enzyme whose catalytic mechanism remains to be elucidated. We titrated that one RLUC molecule can turn over about one hundred molecules of coelenterazine substrate. Mutagenesis of active site residue Pro220 extended the half-life of photon emission, yielding brighter luminescence in *E. coli*. Random mutagenesis uncovered two new mutations that stabilized and increased photon emission *in vivo *and *in vitro*, while ameliorating substrate inhibition. Further amended with a previously identified mutation, a new triple mutant showed a threefold improved k_cat_, as well as elevated luminescence in Arabidopsis. This advances the utility of RLUC as a reporter protein, biosensor, or resonance energy donor.

## Background

*Renilla *luciferase (RLUC) is a cofactor-less, single subunit, blue light emitting luciferase isolated from the marine anthozoan *Renilla reniformis *(RLUC, E.C. number 1.13.12.5, luciferin-2-monooxygenase, decarboxylating) [1]. Aside from its utility as a reporter for gene expression assays, RLUC has also found application in assays for protein interaction based on fragment complementation [2] and bioluminescence resonance energy transfer [3]. The substrate of Renilla luciferase, coelenterazine, consists of a central aromatic imidazopyrazinone, which is derivatized with p-hydroxy-phenyl (R1), benzyl (R2), and p-hydroxy-benzyl (R3) moieties. Using molecular oxygen, RLUC catalyzes an oxidative decarboxylation in which the imidazole ring is opened and carbon dioxide is released [4-7]. Relaxation of the electronically excited coelenteramide reaction product is accompanied by emission of a photon of blue (~470 nm) light.

Compared to the calcium-stimulated photoprotein, aequorin, and its relatives, which utilize the same substrate as RLUC, the catalytic mechanism of RLUC is not yet well understood [8,9]. Photoproteins are single turnover enzymes. Removal of the coelenteramide product and binding of a fresh substrate molecule require a reducing agent and the concomitant removal of calcium [10]. RLUC is not homologous to aequorin but evolved from an α/β hydrolase ancestor closely related to current bacterial dehalogenases. Its structure has recently been solved [11]. Within the large hydrophobic active site, the putative catalytic triad consists of Asp120, His285, and Glu144. Mutagenesis data and inactivation with diethylpyrocarbamate indicate that His285 is important for catalysis, presumably as a general base [12,13]. A model for how coelenterazine and its peroxidized reaction intermediate might be positioned in the active site has been proposed [13].

Re-engineering of the RLUC sequence might ameliorate undesirable properties that arise upon expression in heterologous hosts, which lack RLUCs two partner proteins, a green fluorescent protein and a calcium-responsive coelenterazine binding protein [14,15]. Previous consensus-guided mutagenesis has already led to RLUC versions with improved stability in serum, improved ability to utilize the purple-emitting substrate, bisdeoxycoelenterazine, and altered spectral properties [12,16]. RLUC is well known to be inactivated in the presence of substrate, resulting in most of the light to be emitted as a flash of a few seconds in length. While a short half-life of the enzyme might be beneficial for time-resolved gene expression studies, it is undesirable for protein-interaction studies based on bioluminescence resonance energy transfer [5,17,18].

Here, we describe the results of site directed and random mutagenesis in conjunction with expression and purification of recombinant RLUC enzyme in *E. coli *with the goal of improving specific enzymatic parameters of RLUC. We describe novel mutants with increased k_cat_, extended half-life of photon emission *in vitro *and *in vivo*, and enhanced light emission upon expression in plant cells.

## Methods

### Mutagenesis and other recombinant DNA techniques

The wild type *Renilla reniformis *luciferase cDNA obtained from plasmid pBS-35S-RLUC-attR (Genbank accession, AY995136) [17] was subcloned into the expression vector pET30(a) as an *Nco*I-*Bam*HI fragment, thus adding an N-terminal histidine tag and linker sequence (His-RLUC) [13]. For random mutagenesis, the RLUC cDNA was amplified using an error-prone PCR procedure, GeneMorpho^®^II Random Mutagenesis (Stratagene, La Jolla, CA). A library of 1300 putative mutant clones (strain BL21(DE3)) was grown in LB in white 96-well microtiter plates (Packard, Meriden, CT) to an optical density of about 0.6. Colonies were surveyed for RLUC activity in the presence of 2 μM native coelenterazine (Biotium, Hayward, CA) in the PolarStar plate reader (BMG Labtech, Durham, NC). Candidates with elevated RLUC activity were reconfirmed by inducing RLUC expression at OD = 0.5 with 1 mM IPTG for 1 hour at 30°C and the mutation was identified by DNA sequencing. Subsequently, mutations were also introduced into a human codon-optimized RLUC cDNA (hRLUC, Genbank accession, AAK53368, Packard, Meriden, CT). Site directed mutagenesis was performed using the Quickchange procedure (Stratagene, La Jolla, CA). Mutations were confirmed by resequencing of the entire RLUC coding region to guard against unintended secondary mutations. To generate recombination cloning vectors, the appropriate fragments of pBS-35S-hRLUC-attR (Genbank accession AY995138) and pBS-35S-attR-hRLUC (Genbank accession AY995140) [17] were replaced with the corresponding NheI/BglII restriction fragment from pET30(a)-SuperhRLUC that contained the M185V, K189V, and V267I mutations. The entire SuperhRLUC coding regions of the recombination vectors were confirmed by sequencing.

### Expression and purification of RLUC

RLUC expression in *E. coli *strain BL21(DE3)pLysS was induced with 1 mM IPTG for 3 h hours at 30°C. The accumulation of RLUC in *E. coli *was routinely checked by cell lysis and gel electrophoresis and Coomassie Blue-staining. Mutant proteins generally accumulated to similar levels. RLUC was purified from the soluble cytosolic fraction over a nickel column (His-Bind Kit, Novagen, Darmstadt, Germany) following standard procedures that included sonication, centrifugation of cell debris at 12,000 rpm for 10 minutes at 4°C, and filtration of the supernatant through a 0.45 micron filter to prevent clogging of resin. Protein was affinity purified according to the manufacturer's protocol and eluted with 1 M imidazole, 0.5 M NaCl, 20 mM Tris-HCl, pH, 7.9. After elution, RLUC was dialyzed overnight against 2 L of phosphate buffered saline (PBS, pH7.2) in order to remove imidazole. Protein concentration was determined using the BCA assay (Pierce, Rockford, IL) with BSA as a standard. Alternatively, the protein concentration of preparations that were free of imidazole was measured by UV-absorbance using an extinction coefficient of 65,040 M^-1 ^cm^-1 ^[19]. Purified RLUC protein was stored in PBS with 50% glycerol at -70°C in small aliquots or stored at 4°C for up to 2 weeks.

### Kinetics of RLUC enzyme activity

Enzyme assays were conducted using freshly purified RLUC enzyme at a concentration of 1 nM or as otherwise indicated in 1 ml PBS (pH 7.2). Native coelenterazine substrate was added from a 250× stock solution of the indicated concentration in ethanol (final ethanol concentration, 0.8%), the solution was mixed by tapping to ensure a maximal supply of oxygen, and the luminescence activity was recorded in the TD20/20 luminometer (Turnerdesigns, Sunnyvale, CA). The first 5-second luminescence reading was taken as a measure of enzyme activity. The K_M _values of wild type RLUC and selected mutants were calculated according to standard Michaelis-Menten theory using Prism software (GraphPad Software Inc., San Diego, CA) from at least 3 repeat measurements. Several independent protein preparations yielded similar K_M _values.

### Emission spectra

Luminescence spectra were recorded under the same condition as the enzyme assay using a spectroluminometer (Photon Technology International, Inc., Birmingham, NJ), except that the assay volume was 2 ml. Native coelenterazine substrate was 2 μM (ethanol concentration, 0.8%). Protein concentration was 10 nM purified enzyme or as otherwise indicated. Generally, the emission spectrum was analyzed with the Felix32 software (Photon Technology International, Inc., Birmingham, NJ). All spectra were recorded at 1 nm per second from short to long wavelength. No adjustments for detector sensitivity or luminescence decay over time were made; nevertheless, the spectra are directly comparable among each other and emphasize the differences in emission in the short-wavelength region.

### Substrate/enzyme titration

The amount of RLUC enzyme needed to deplete a nearly saturating amount of substrate (0.1 ml of 1 μM or 10 μM coelenterazine) was determined by titration. Parallel reactions were set up with RLUC at concentrations between 100 nM and 1 nM in 100 μl of assay buffer (50 mM potassium phosphate, pH 7.4, 500 mM NaCl, 1 mM EDTA) [5]. Reactions were allowed to proceed for at least 2 hours until luminescence had decayed to near-background levels. Each spent reaction was then split into two aliquots. To one aliquot fresh enzyme (10 nM) was added. If no increase in luminescence was observed it was concluded that the substrate must have been used up completely allowing us to deduce the stoichiometry between enzyme and substrate. The second 50 μl aliquot was supplemented with 10 μM substrate to check whether the substrate had been used up completely by the enzyme or whether RLUC activity had been depleted.

### Spectrophotometric assay of RLUC activity

The spectrum of a 10 μM solution of coelenterazine in assay buffer was recorded. RLUC was added to 100 nM and the spectrum re-recorded after 10 minutes and 60 minutes of reaction time. The extent of spontaneous degradation of coelenterazine was determined in a control reaction without added enzyme.

### Plant growth condition and transgenic lines

Columbia wild type and transgenic seedlings were germinated on 0.8% agar medium containing Murashige and Skoog salts (MS; Sigma, St. Louis) and 1% sucrose without antibiotics. The transgenic plants expressing the hRLUC or SuperhRLUC cassettes were grown on a MS selection media containing 1.5 mg/l ammonium glufosinate herbicide (Basta; Sigma, St. Louis).

### Measurement of Renilla luciferase activity in transgenic Arabidopsis

Luminescence units were measured from 10 day-old seedlings in the presence of 2 μM coelenterazine (Biotium, Hayward, CA) in water using a TD-20/20 tube luminometer that is equipped with the blue-color filter. After adding 2 μM coelenterazine, samples were incubated in darkness for 10 min at room temperature to allow the substrate to penetrate into plants and to allow delayed chlorophyll autofluorescence to decay [17].

### Protein extraction from plants and western blotting

Plant protein was extracted with passive lysis buffer from the Dual-Luciferase^® ^Reporter Assay System (Promega, Madison, WI) and RLUC detected by western blotting using a monoclonal antibody (Chemicon, Temecula, CA).

### Photon-counting images

Transgenic 7–10 days old *Arabidopsis *seedlings were imaged on a Nikon microscope with a Hamamatsu C2400 ICCD (Meyer Instruments, Houston, TX). Seedlings were pre-incubated in 2 μM coelenterazine for 5 min and then photon emission was recorded for 5 min. Images were pseudo-colored with ImageJ (NIH, Bethesda, MD).

## Results and discussion

**Figure 1:**
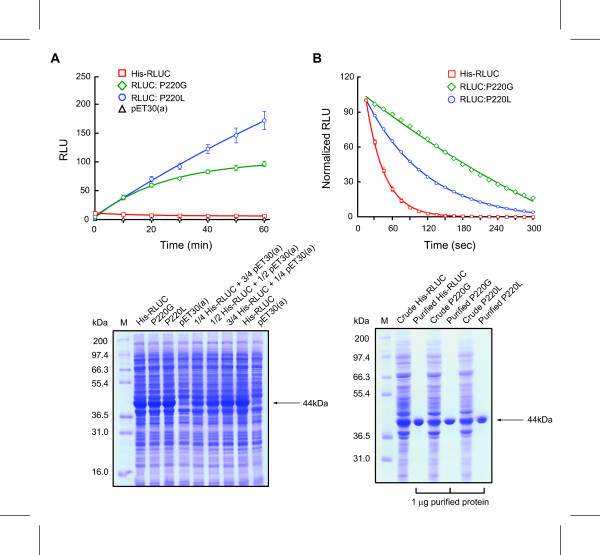
**Half-life of RLUC enzyme activity. **(A) Time course of *in vivo *luminescence in *E. coli *cells expressing wild type RLUC and two P220 mutants. (B) Time course of *in vitro *luminescence of wild type RLUC and two P220 mutants. Enzyme and substrate concentrations were 10 nM and 2 μM respectively in PBS buffer pH7.2. Absolute activities were normalized for better comparison (first time-point = 100). Results shown are averages from three biological replicates each performed in triplicate. The Coomassie stained SDS-PAGE gels below show that the P220 mutants and wild-type RLUC accumulate to equivalent levels in E. coli. RLU stands for relative luminescence units.

Previous docking simulations and mutagenesis of RLUC active site residues D120, E144, and H285, which are conserved with the catalytic triad of the dehalogenase LinB, suggested that they also underlie the catalytic mechanism of RLUC. Meanwhile, two other residues, N53 and W121, were proposed to function in binding to the R1 ring of coelenterazine [13]. When the neighboring P220 was mutated to ten different residues, several mutants displayed the unusual characteristic that *E. coli in vivo *luminescence was initially low, but rose dramatically over time, soon surpassing the peak luminescence of cells expressing wild-type RLUC (Figure 1A) [13]. When tested *in vitro *after purification, the P220G and P220L mutant proteins showed only 4% and 16% of the initial luciferase activity of wild-type RLUC, respectively (not shown). However, compared to wild-type RLUC and many other mutants tested, whose half-life of photon emission *in vitro *was about 40 seconds, P220G and P220L yielded a strikingly more stable luminescence output over time (Figure 1B).

**Figure 2:**
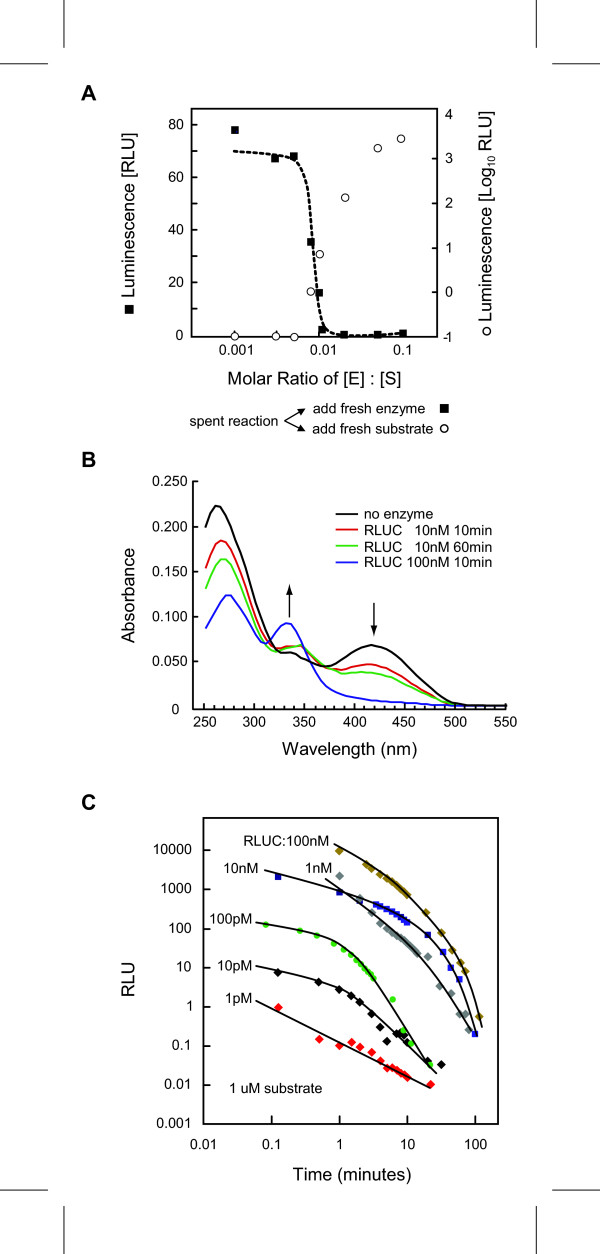
**Decay properties of RLUC enzyme activity.** (A) A titration assay indicates that 1 molecule of RLUC can turn over ~100 molecules of coelenterazine. A 1 μM solution of native coelenterazine substrate was incubated with increasing amounts of RLUC enzyme (1 nM to 100 nM) and the reaction was allowed to go to completion. Once light emission had ceased, the reaction was split in half and supplemented with fresh enzyme (squares) to examine whether all substrate had been depleted; or with fresh substrate (circles) to check for residual enzyme activity. Note, if an excess of substrate was added, the loss of RLUC activity was not reversible by adding fresh substrate. (B) Spectrophotometric confirmation of substrate turnover by RLUC. Absorbance spectra were recorded for 10 μM coelenterazine before (black trace) and after (blue trace) turnover by 100 nM RLUC. Two traces of partially completed reactions are shown for comparison (red and green traces). (C) Half life of RLUC activity in the *in vitro *assay as measured by luminescence decay kinetics.

It is unclear why RLUC rapidly loses its enzymatic activity when in contact with substrate. The turnover number of RLUC is low (111 μmol min^-1 ^μmol^-1 ^enzyme) [5] but the sum total of substrate molecules turned over by one molecule of RLUC has not been reported. The loss of luminescence activity cannot be attributed to oxygen depletion because the concentration of dissolved oxygen in water (280 μM at 20°C) is much higher than that needed to react with coelenterazine (1 μM). By titrating the amount of wild-type enzyme needed to deplete the luminescence potential of a solution of coelenterazine substrate, we determined that one molecule of enzyme is able to turn over up to 100 molecules of native coelenterazine (Figure 2A). To confirm that a sufficient amount of RLUC causes complete turnover of the substrate, the enzymatic reaction was also followed by measuring absorbance of coelenterazine at 420 nm (Figure 2B). The vast majority of photons are emitted within the first ten minutes of the reaction (Figure 1B), which coincides with the drop in coelenterazine absorbance at 420 nm and appearance of a new peak at 350 nm, which is attributed to coelenteramide. Therefore, the drop in luminescence cannot simply be explained by a drop in quantum efficiency. Coelenteramide is a strong competitive inhibitor of RLUC (K_I _~ 23 nM) [6], and its accumulation must contribute to the loss of activity. However, it cannot be solely responsible, because enzyme activity is also lost at enzyme concentrations as low as 1 pM, when, in light of the titration data, the concentration of accumulated coelenteramide is far below the K_I _(Figure 2C).

Next, we screened RLUC mutants generated by random mutagenesis for mutants with increased peak of light emission in *E. coli *or increased stability of light emission over time. Upon DNA sequencing and protein purification, the activities of two mutants, V267I and K189V, were elevated over wild-type RLUC (Table 1). A third mutant, M185G, was of interest because of a slightly increased half life (not shown), although k_cat_was decreased. While K189 and M185 lie in the presumptive gateway that guides the substrate into the active site [11], V267 lies outside of the active site, as do most of the eight mutations constituting the RLUC8 mutant, which has an increased k_cat _[12].

**Table 1:** Activities of RLUC mutants selected for improved enzymatic activity.

Mutations	Name	Activity ± SD	Condition
Native RLUC cDNA			

none	His-RLUC	100	*in vitro *^1)^
V267I	-	163 ± 33	*in vitro*
K189V	-	128 ± 16	*in vitro*
K189V+V267I	RLUC+	317 ± 82	*in vitro*
M185V+K189V+V267I	SuperRLUC	411 ± 113	*in vitro*

Codon-optimized RLUC cDNA (hRLUC)			

none	His-hRLUC	100	*in vivo *^2)^
K189V	-	175 ± 70	*in vivo*
M185V+K189V	-	425 ± 120	*in vivo*
M185V+K189V+V267I	SuperhRLUC	475 ± 130	*in vivo*

^1) ^*In vitro *activites are initial luminescence values upon addition of substrate (2 μM coelenterazine). The unmutated version was set to 100.

^2) ^*In vivo *activities are luminescence activities from *E. coli *strain BL21 without IPTG induction (2 μM coelenterazine).

The coelenteramide reaction product relaxes from the excited state to the ground state under emission of a photon of light. The precise peak wavelength of light emission is influenced by the protonation state of the reaction product and is also dependent on the physical environment (hydrophobicity) in the active site [20]. The emission spectra of K189V and M185G were similar to wild-type RLUC (not shown). In contrast, V267I showed two major peaks, a blue-shifted shoulder of 390 nm, indicative of the formation of a neutral coelenteramide [7,21-23] and a blue-shifted maximum at 450 nm [see Additional file 1].

**Figure 3:**
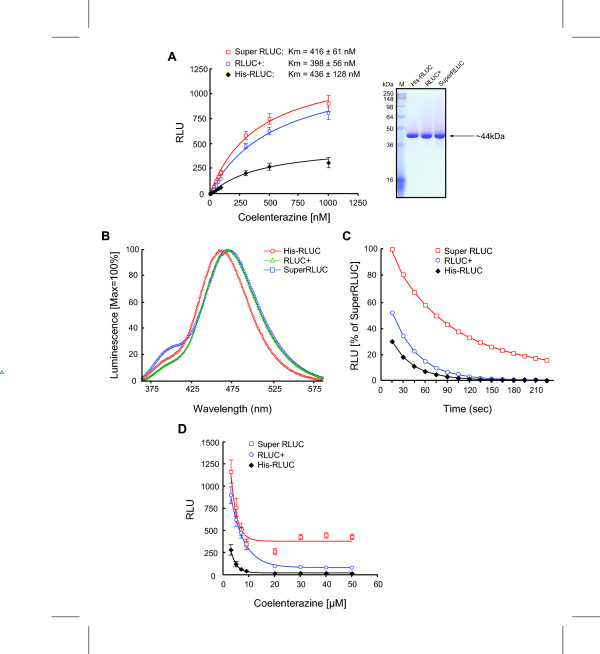
**Enzyme kinetics of optimized RLUC proteins.** (A) Derivation of K_M _values. The K_M _for wild-type RLUC was similar to previously published data such as 210 nM (coelenterazine h)^5 ^and 300 nM^23^. Also shown are wild-type RLUC, RLUC+ and SuperRLUC, which were purified by nickel affinity chromatography and run on a polyacrylamide gel. (B) Luminescence spectra for RLUC, RLUC+ and Super-RLUC. (C) Time course of luminescence activity. Note the increased half life of activity of the RLUC+ and SuperRLUC mutants. (D) Inhibition of RLUC activity by high substrate concentration.

The putative beneficial mutations, K189V and V267I, were combined into a double mutant, named RLUC+. The two mutations appeared to act additively, yielding a more than two-fold increase in the apparent k_cat _of light emission compared to wild-type RLUC (Figure 3A). From another set of RLUC mutations that yielded increased luminescence in mammalian cells [12,24], we selected M185V, a mutation that increased the stability of enzyme activity in serum and the ability to luminesce using the substrate bis-deoxycoelenterazine [12]. Its incorporation into RLUC+ yielded a slight further increase in light emission (SuperRLUC; Figure 3A). Including the P220G mutation provided no further enhancement (not shown). Neither RLUC+ nor SuperRLUC had an altered K_M _and their emission spectra were similar to wild type (Figure 3A and 3B). Interestingly, the K189V mutation suppressed the blue shift and the marked purple emission detected in the V267I mutant. However, the luminescence of SuperRLUC had a two-fold longer half-life *in vitro *compared to wild-type RLUC (Figure 3C). RLUC is known to be inhibited by aggregation at high concentrations of substrate (above 3 μM) [6]. RLUC+ and SuperRLUC were less sensitive to substrate inhibition (Figure 3D). Elevated RLUC activity was also observed when the mutations were introduced into a codon-optimized cDNA (hRLUC, Table 1).

**Figure 4:**
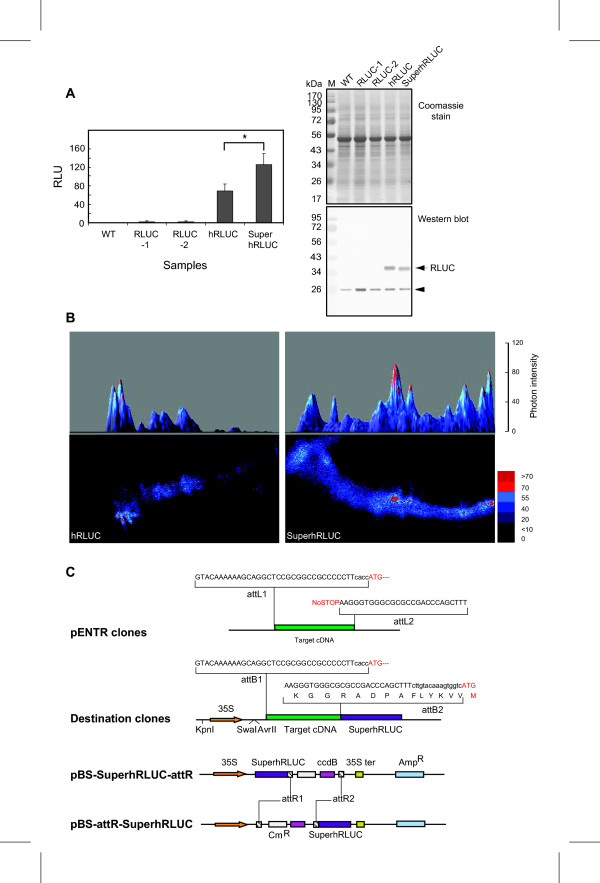
**Improvement of light emission by SuperhRLUC in Arabidopsis and recombination vectors.** (A) *In vivo *luminescence measurement of transgenic *Arabidopsis *expressing regular RLUC (2 lines), hRLUC, or SuperhRLUC. Asterisk represents the significant increase of luciferase activity of SuperhRLUC over hRLUC (P < 0.01, two tailed t-test; n = 4 repeats with 25 seedlings each). The immunoblot below probed with RLUC antibody confirms that hRLUC and SuperhRLUC (36 kDa) accumulate to similar, high, levels; the original RLUC can only be detected on the immunoblot after prolonged development. The arrowhead indicates a non-specific immunoreaction. (B) Photon-counting images of representative seedling roots. Photon emission in the primary roots of SuperhRLUC transgenic Arabidopsis was stronger compared to regular hRLUC (lower panels). The upper panels show a 3-dimensional version of the images below in which photon intensity is encoded in the third axis. Seedlings were incubated in 2 μM coelenterazine and imaged for 5 min. (C) pBS-SuperhRLUC-attR and pBS-attR-SuperhRLUC are recombination vectors for expression of SuperhRLUC fusion proteins, which contain the lambda *att *recombination sites utilized by the Gateway™ (Invitrogen) system [25]. Sequence elements flanking an insert (target cDNA) are shown before (pENTR) and after (Destination) attL × attR recombination. The 35S indicates a strong promoter in plants, which can be replaced by restriction digestion with KpnI and SwaI or AvrII. The ccdB gene provides for counter-selection of non-recombinants.

SuperhRLUC gave rise to approximately two-fold higher luminescence values than regular hRLUC when expressed in stably transgenic Arabidopsis seedlings (Figure 4A and 4B). In addition, SuperhRLUC protein extracted from plants had a prolonged half life of photon emission [see Additional file 2]. For easy construction of SuperhRLUC fusion proteins, new recombination cloning vectors harboring SuperhRLUC were generated (Figure 4C); these are available from the corresponding author upon request. The improved luciferase activity and stability of SuperhRLUC in Arabidopsis should be beneficial for utilization of RLUC as a reporter protein or biosensor. Why RLUC suffers from irreversible inactivation of its enzymatic activity still requires further study. Dramatic improvements in luminescence emission can be expected if this limitation can be resolved.

## Conclusion

Mutant versions of *Renilla *luciferase with increased k_cat _were identified from a library of random mutations expressed in E. coli. A combination of two or three mutations resulted in increased activity of the Renilla luciferase reporter enzyme in transgenic Arabidopsis.

## Abbreviations

RLU: relative light units; RLUC: Renilla luciferase.

## Competing interests

The authors declare that they have no competing interests. All authors read and approved the final manuscript.

## Authors' contributions

JW and AGV designed experiments and wrote the manuscript. JW performed all experiments except those in Figure 2, which were performed by AGV.

## Supplementary Material

Additional file 1 Luminescence spectra. Luminescence emission scans of selected single mutants with improved enzymatic properties. Click here for file

Additional file 2 Enzyme stability measurements. Enzyme stabilities of RLUC, hRLUC, and SuperhRLUC after extraction from transgenic *Arabidopsis *(10 μM coelenterazine; n = 4; average ± s.d.). Click here for file
