# Supplementary figures and images for: Mutational optimization of the coelenterazine-dependent luciferase from Renilla
Source: Plant Methods. 2008 Sep 30;4:23. doi: 10.1186/1746-4811-4-23 (PMC2565673; doi:10.1186/1746-4811-4-23)

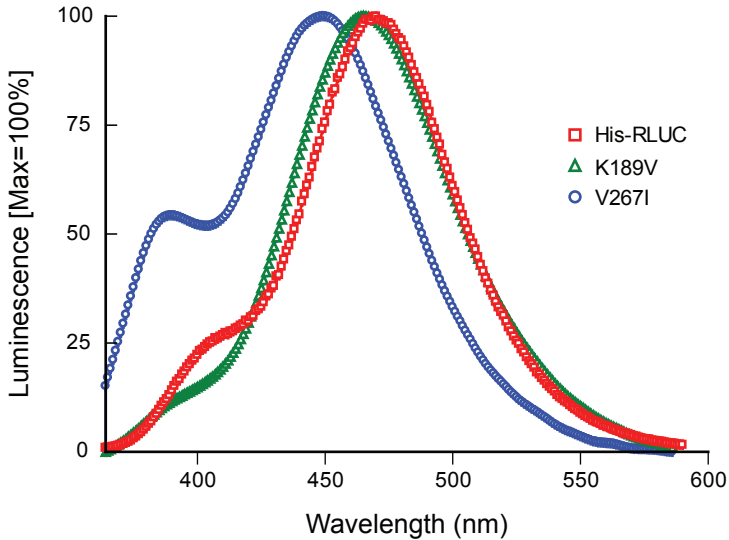

Supplement: Additional file 1 — Luminescence spectra. Luminescence emission scans of selected single mutants with improved enzymatic properties. [file 1746-4811-4-23-S1.pdf]

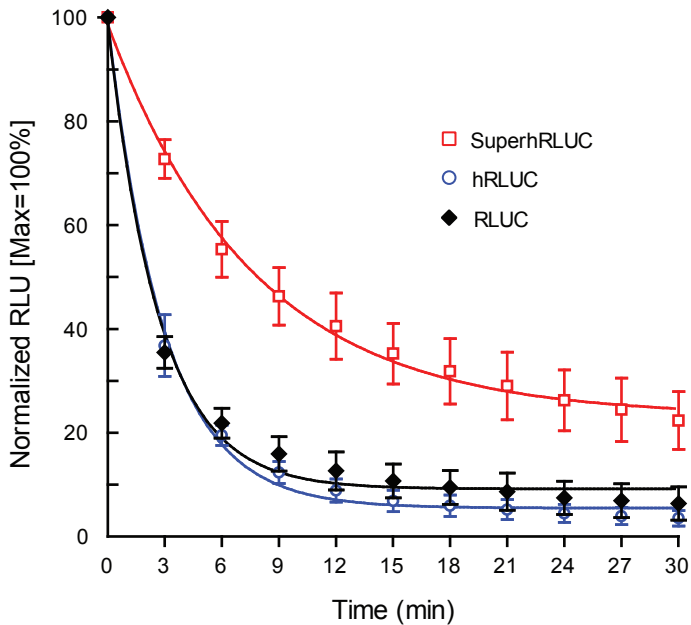

Supplement: Additional file 2 — Enzyme stability measurements. Enzyme stabilities of RLUC, hRLUC, and SuperhRLUC after extraction from transgenic Arabidopsis (10 μM coelenterazine; n = 4; average ± s.d.). [file 1746-4811-4-23-S2.pdf]
